# Azithromycin-loaded PLGA microspheres coated with silk fibroin ameliorate inflammation and promote periodontal tissue regeneration

**DOI:** 10.1093/rb/rbae146

**Published:** 2024-12-14

**Authors:** Zhaoguang Ouyang, Xiaoyu Chen, Zhengyang Wang, Yue Xu, Zhe Deng, Liangyu Xing, Li Zhang, Meilin Hu, Haocong Li, Tengye Lian, Feng Gao, Chunyi Liu, Yangyang Zhou, Lu Sun, Ying ChengYao Wang, Dayong Liu

**Affiliations:** Department of Endodontics, Tianjin Medical University School and Hospital of Stomatology & Tianjin Key Laboratory of Oral Soft and Hard Tissues Restoration and Regeneration, Tianjin 300070, PR China; Tianjin Medical University Institute of Stomatology, Tianjin 300070, PR China; Department of Preventive Dentistry, School and Hospital of Stomatology, Guangdong Engineering Research Center of Oral Restoration and Reconstruction & Guangzhou Key Laboratory of Basic and Applied Research of Oral Regenerative Medicine, Guangzhou Medical University, Guangzhou 510013, PR China; Department of Endodontics, Tianjin Medical University School and Hospital of Stomatology & Tianjin Key Laboratory of Oral Soft and Hard Tissues Restoration and Regeneration, Tianjin 300070, PR China; Tianjin Medical University Institute of Stomatology, Tianjin 300070, PR China; Department of Endodontics, Tianjin Medical University School and Hospital of Stomatology & Tianjin Key Laboratory of Oral Soft and Hard Tissues Restoration and Regeneration, Tianjin 300070, PR China; Tianjin Medical University Institute of Stomatology, Tianjin 300070, PR China; Department of Endodontics, Tianjin Medical University School and Hospital of Stomatology & Tianjin Key Laboratory of Oral Soft and Hard Tissues Restoration and Regeneration, Tianjin 300070, PR China; Tianjin Medical University Institute of Stomatology, Tianjin 300070, PR China; College of Integrated Chinese and Western Medicine, Hunan University of Chinese Medicine, Changsha, Hunan 410208, PR China; Sidney Kimmel Comprehensive Cancer Center at Johns Hopkins, Baltimore, MA 21205, USA; Department of Endodontics, Tianjin Medical University School and Hospital of Stomatology & Tianjin Key Laboratory of Oral Soft and Hard Tissues Restoration and Regeneration, Tianjin 300070, PR China; Tianjin Medical University Institute of Stomatology, Tianjin 300070, PR China; Department of Endodontics, Tianjin Medical University School and Hospital of Stomatology & Tianjin Key Laboratory of Oral Soft and Hard Tissues Restoration and Regeneration, Tianjin 300070, PR China; Tianjin Medical University Institute of Stomatology, Tianjin 300070, PR China; Department of Endodontics, Tianjin Medical University School and Hospital of Stomatology & Tianjin Key Laboratory of Oral Soft and Hard Tissues Restoration and Regeneration, Tianjin 300070, PR China; Tianjin Medical University Institute of Stomatology, Tianjin 300070, PR China; Department of Endodontics, Tianjin Medical University School and Hospital of Stomatology & Tianjin Key Laboratory of Oral Soft and Hard Tissues Restoration and Regeneration, Tianjin 300070, PR China; Tianjin Medical University Institute of Stomatology, Tianjin 300070, PR China; Department of Endodontics, Tianjin Medical University School and Hospital of Stomatology & Tianjin Key Laboratory of Oral Soft and Hard Tissues Restoration and Regeneration, Tianjin 300070, PR China; Tianjin Medical University Institute of Stomatology, Tianjin 300070, PR China; Department of Endodontics, Tianjin Medical University School and Hospital of Stomatology & Tianjin Key Laboratory of Oral Soft and Hard Tissues Restoration and Regeneration, Tianjin 300070, PR China; Tianjin Medical University Institute of Stomatology, Tianjin 300070, PR China; Department of Endodontics, Tianjin Medical University School and Hospital of Stomatology & Tianjin Key Laboratory of Oral Soft and Hard Tissues Restoration and Regeneration, Tianjin 300070, PR China; Tianjin Medical University Institute of Stomatology, Tianjin 300070, PR China; Department of Endodontics, Tianjin Medical University School and Hospital of Stomatology & Tianjin Key Laboratory of Oral Soft and Hard Tissues Restoration and Regeneration, Tianjin 300070, PR China; Tianjin Medical University Institute of Stomatology, Tianjin 300070, PR China; Department of Periodontics and Oral Medicine, University of Michigan School of Dentistry, Ann Arbor, MI 48105, USA; Periodontal and Implant Microsurgery Academy (PiMA), University of Michigan School of Dentistry, Ann Arbor, MI 48105, USA; Department of Operative Dentistry and Endodontics, Tianjin Stomatological Hospital, School of Medicine, Nankai University, Tianjin 300041, PR China; Tianjin Key Laboratory of Oral and Maxillofacial Function Reconstruction, Tianjin 300041, PR China; Department of Endodontics, Tianjin Medical University School and Hospital of Stomatology & Tianjin Key Laboratory of Oral Soft and Hard Tissues Restoration and Regeneration, Tianjin 300070, PR China; Tianjin Medical University Institute of Stomatology, Tianjin 300070, PR China; School and Hospital of Stomatology, Hebei Medical University & Hebei Key Laboratory of Stomatology & Hebei Clinical Research Center for Oral Diseases, Shijiazhuang, Hebei 050011, PR China

**Keywords:** periodontitis, azithromycin, PLGA microspheres, silk fibroin, periodontal tissue regeneration

## Abstract

Periodontitis, a widespread inflammatory disease, is the major cause of tooth loss in adults. While mechanical periodontal therapy benefits the periodontal disease treatment, adjunctive periodontal therapy is also necessary. Topically applied anti-inflammatory agents have gained considerable attention in periodontitis therapy. Although azithromycin (AZM) possesses excellent anti-inflammatory properties, its bioavailability is limited owing to poor water solubility and the absence of sustained release mechanisms. Herein, we synthesized biodegradable microspheres (AZM@PLGA-SF) for sustained AZM release to locally ameliorate periodontal inflammation and facilitate periodontal tissue regeneration. AZM was encapsulated in poly (lactic-co-glycolic acid) (PLGA) microspheres (AZM@PLGA) using single emulsion-solvent evaporation, followed by surface coating with silk fibroin (SF) via electrostatic adsorption, reducing the initial burst release of AZM. *In vivo*, local treatment with AZM@PLGA-SF microspheres significantly reduced periodontal inflammation and restored periodontal tissue to healthy levels. Mechanically, the formulated microspheres regulated the periodontal inflammatory microenvironment by reducing the levels of pro-inflammatory cytokines (tumor necrosis factor -α, interleukin [IL]-6, interferon-γ, IL-2, and IL-17A) in gingival crevicular fluid and promoted the expression of anti-inflammatory cytokines (IL-4 and IL-10). AZM@PLGA-SF microspheres demonstrated excellent biological safety. Therefore, we introduce an anti-inflammatory therapy for periodontitis with substantial potential for mitigating periodontal inflammation and encouraging the repair and regeneration of periodontal tissues.

## Graphical abstract

**Graphical Abstract:**
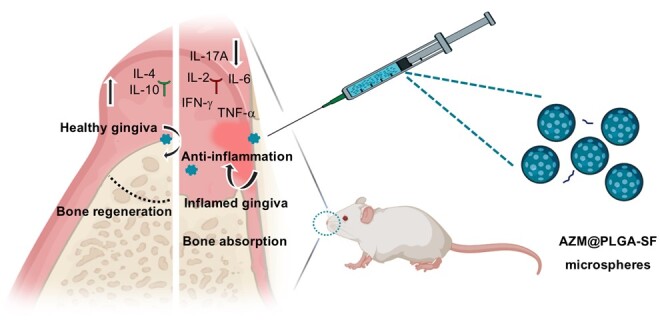

## Introduction

Periodontitis is a chronic multifactorial inflammatory disease associated with dysbiotic plaque biofilms and characterized by progressive destruction of the tooth-supporting apparatus. Meanwhile, periodontal infection may impact extraoral health and there is biological link between periodontitis and systemic outcomes [1–4]. Aberrant host–bacteria interaction contributes to the continuous inflammatory immune response that results in cytokine cascades and secondary bone loss. While appropriate inflammation is crucial for initiating rehabilitation progress, uncontrollable inflammation causes extensive tissue damage and delayed healing [5]. Therefore, it is crucial to modulate the host’s inflammatory response. Currently, mechanical therapy is an essential component of periodontal treatment. Using anti-inflammatory agents as therapeutic strategies additional to conventional mechanical treatment can help regulate the host response and promote periodontal tissue regeneration [3, 6]. Metalloproteinase inhibitors such as doxycycline and non-steroidal anti-inflammatory drugs such as flurbiprofen have been used to mitigate inflammation and prevent alveolar bone destruction in periodontitis [7]. However, these treatments are limited owing to adverse side effects and unpredictable efficacy [8, 9]. Azithromycin (AZM), recognized for its potent anti-inflammatory properties under various conditions [10, 11], has been shown to modulate inflammatory cytokine expression and reduce alveolar bone destruction in periodontitis [12, 13]. Our previous *in vitro* study demonstrated the ability of AZM to promote osteogenic differentiation of periodontal ligament stem cells in an inflammatory microenvironment, suggesting its potential to promote periodontal tissue regeneration [14]. Nevertheless, systemic administration of AZM is associated with potential issues such as poor biodistribution, gastrointestinal intolerance and the need for administering high doses [15]. Conversely, topical application of AZM can reduce systemic side effects. However, the topical application of AZM formulations faces challenges due to their inherent hydrophobicity and limited water solubility for bioavailability [7, 16]. Additionally, drug delivery in the oral cavity is challenged by factors such as saliva and gingival crevicular fluid secretion, tongue movement and poor local retention [17]. Therefore, it is crucial to select a suitable carrier for topical AZM delivery to achieve higher efficacy in periodontitis treatment.

Poly(lactic-co-glycolic acid) (PLGA) microspheres are extensively employed as drug delivery vehicles owing to their excellent biocompatibility, biodegradability and convenient administration properties [18, 19]. However, PLGA lacks favorable hydrophilicity and cell attachment sites [20]. Additionally, most drug-loaded PLGA microspheres exhibit an initial uncontrolled drug burst release, which may lead to potential toxicity and reduced therapeutic efficacy [21]. While studies have reported on the use of AZM-loaded PLGA for the topical treatment of periodontitis [22, 23], few researches addressed the modification of PLGA surface to control AZM release. Moreover, there is limited literatures focusing on the anti-inflammatory properties of AZM specifically in the topical treatment of periodontitis. Silk fibroin (SF) is a natural protein with exceptional biocompatibility, biodegradability and mucosal adhesive properties [24]. SF has been shown to enhance the hydrophilicity and mucosal adhesive properties of the PLGA matrix and aid in retarding drug release [25, 26]. Therefore, combining PLGA microspheres with SF can be suitable for loading AZM to treat periodontitis topically.

**Figure 1.**
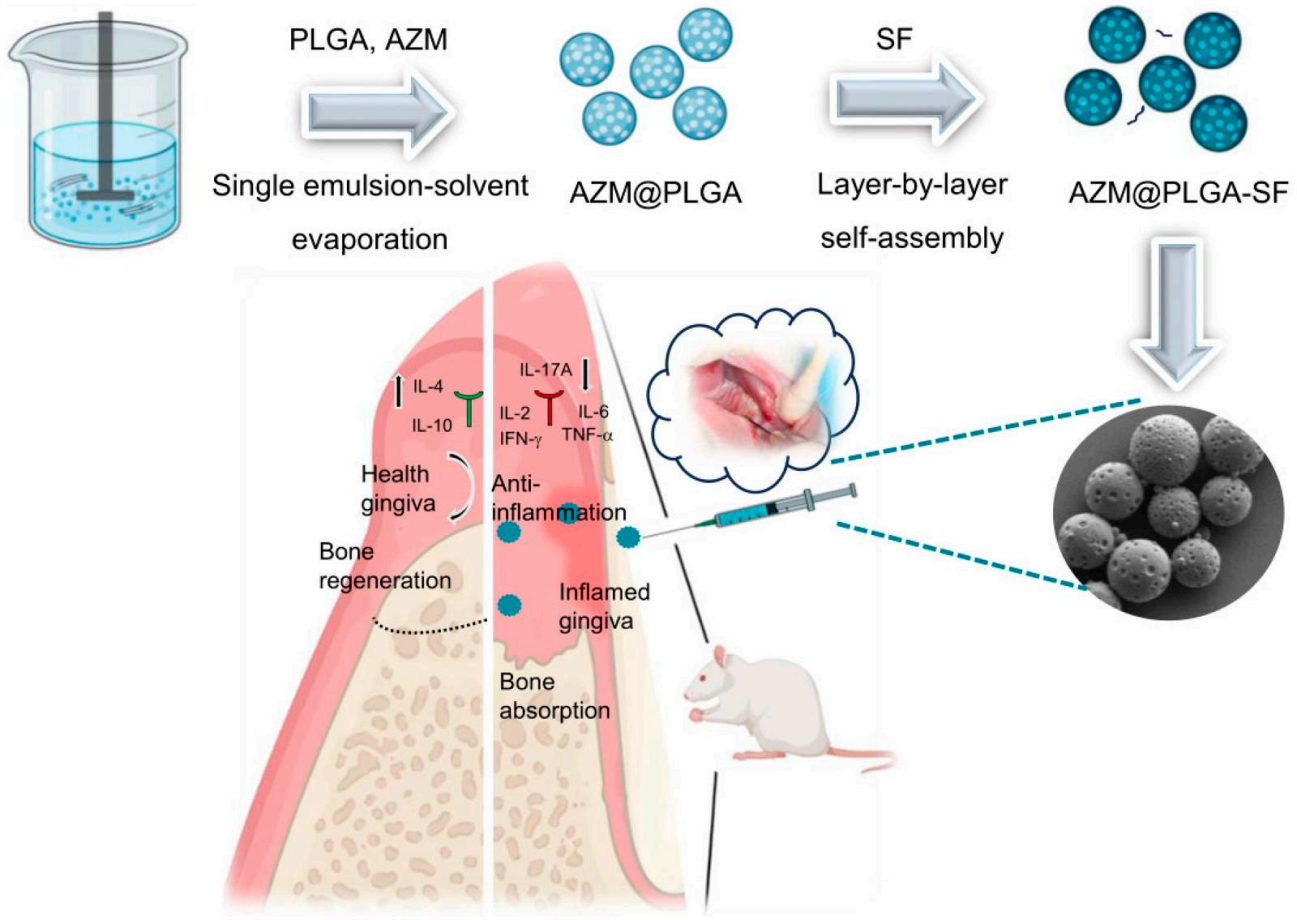
Schematic illustration for the preparation and therapeutic efficiency of AZM@PLGA-SF microspheres for periodontitis treatment. The microspheres ameliorate periodontal inflammation and promote alveolar bone regeneration. We thank Biorender (www.biorender.com) for its graphical assistance.

As shown in Figure 1, AZM-loaded PLGA microspheres coated with SF (AZM@PLGA-SF) were developed to ameliorate periodontal inflammation and promote periodontal tissue regeneration. AZM@PLGA-SF microspheres were synthesized using a single emulsion-solvent evaporation technique, followed by the alternate deposition of polyallylamine hydrochloride (PAH) and SF *via* electrostatic force-driven layer-by-layer (LbL) assembly [26–28]. The formulated microspheres were subsequently characterized. Moreover, the *in vivo* therapeutic efficacy and underlying mechanisms were assessed using an experimental periodontitis rat model. Overall, AZM@PLGA-SF microspheres were successfully synthesized, exhibiting sustained release of AZM, substantial anti-inflammatory effects and promoting periodontal tissue regeneration. Consequently, AZM@PLGA-SF microspheres offer a promising therapeutic approach for the treatment of periodontitis.

## Materials and methods

### Materials

PLGA (MW 50 000; 50:50) was procured from Jinan Daigang Biomaterial Co., Ltd (Shandong, China). Phosphate-buffered saline (PBS) and raw AZM were obtained from Beijing Solarbio Science & Technology Co. Ltd (Beijing, China). Poly(vinyl alcohol) (PVA) (degree of polymerization, 500; degree of hydrolysis, 88%) was provided by Sinopec Sichuan Vinylon Works (Sichuan, China). PAH (MW ≈ 17 500) was acquired from Sigma-Aldrich, Inc., USA. SF was sourced from Beijing Sinolactide Medical Technology Company (Beijing, China). All other analytical grade chemical reagents were used as received, without any modifications. The ultrapure water used in all experiments was generated using a Milli-Q synthesis system (Millipore Corp., USA).

### Synthesis of AZM@PLGA-SF microspheres

AZM@PLGA-SF microspheres were synthesized using the oil-in-water (O/W) emulsion-solvent evaporation method, combined with layer-by-layer self-assembly [26–28]. Initially, 100 mg of PLGA and a defined quantity of AZM were dissolved in 10 ml of a methylene chloride solution. This organic phase was subsequently introduced dropwise into 50 ml of an aqueous solution containing 1% PVA while continuously stirring at 800 rpm for 2 h to ensure the complete evaporation of methylene chloride. The formed microspheres were isolated by centrifugation, washed three times with ultrapure water and freeze-dried. For surface modification, the microspheres were first incubated with PAH, followed by incubation with SF aqueous solutions (2 mg/ml in 0.5 M NaCl), allowing alternate adsorption of these substances onto the microsphere surfaces. To facilitate crosslinking, the microspheres were subsequently immersed for 1 h in a 2% (w/v) glutaraldehyde solution, prepared under appropriate safety measures. After this step, the crosslinking reaction was quenched by adding sodium borohydride (NaBH_4_, 3 × 10^−2^ M) for 30 min. Finally, the AZM@PLGA-SF microspheres were subjected to three additional washes with ultrapure water to eliminate any residual chemicals and collected by centrifugation.

### Physical characterizations of microspheres

The surface morphologies of raw AZM and microspheres were characterized using scanning electron microscopy (SEM) (Carl Zeiss Merlin Compact, Baden-Wurttemberg, Germany). A droplet of the microsphere suspension was carefully placed onto a silicon wafer, which was then allowed to air-dry at ambient room temperature prior to examination. After drying, the samples were sputter-coated with gold using an ion coater (ETD-2000C) to enhance their electrical conductivity. SEM observations were conducted at an accelerating voltage of 3.0 kV to optimize resolution and contrast. For particle size analysis, the diameters of 100 randomly selected microspheres from the SEM images were measured using Nano Measurer software. Subsequently, the measured diameters were statistically analyzed to calculate the average size and assess the size distribution of the microspheres.

Microsphere sample (20 mg) was dispersed in 2 ml of PBS solution to evaluate its injectability. The resulting suspension was loaded into 1 ml syringes then injected into a plastic petri dish using a 34G (0.18 × 8 mm) narrow needle.

An X-ray powder diffractometer (Rigaku Ultima IV) equipped with Cu Kα radiation (*λ* = 1.54178 Å) over the 2*θ* range of 5°−60° was employed to characterize the crystallinity of samples. The chemical composition of samples was determined by Fourier transform infrared spectrometry (FTIR). For IR, samples were mixed with KBr, punched and the spectrum was recorded using FTIR spectrometer (Nicolet iS10, Thermo Fisher Scientific, Waltham, USA) in the transmission mode.

### Assessment of SF deposition on the microsphere surface

The microsphere samples were incubated in the dark with fluorescein isothiocyanate (FITC)-labeled PAH aqueous solutions (0.5 M NaCl solution) for 30 min, followed by three rinses with 0.5 M NaCl solution to remove unbound entities. The microspheres were then redispersed in ultrapure water for subsequent analysis. Fluorescence imaging was performed using a confocal laser-scanning microscope (CLSM; Leica TCS SP8, Wetzlar, Germany) at an excitation wavelength of 488 nm. Additionally, dynamic light scattering (DLS; Malvern Zetasizer Nano ZS, Malvern Instruments, UK) was performed to measure the zeta potential of PAH/SF multilayer-coated AZM@PLGA microspheres before and after the crosslinking process.

### Drug loading and encapsulation efficiency of AZM@PLGA-SF microspheres

Initially, a standard curve was established for AZM. Specifically, 10 mg of AZM was dissolved in anhydrous ethanol and diluted in PBS to pH 6.8. Subsequently, the AZM solution was mixed with 75% (v/v) sulfuric acid and allowed to react for 30 min. The resulting solution was subjected to AZM content analysis using a UV–visible spectrophotometer at a maximum absorption wavelength of 482 nm [29]. The calibration curve exhibited linearity within the AZM concentration range of 7.5–30 µg/ml, presenting a correlation coefficient (*R*^2^) of 0.9997 (*y* = 0.0317*x* − 0.0076). To assess the drug loading and encapsulation efficiency of the AZM@PLGA-SF microspheres, approximately 10 mg of microspheres were dissolved in an appropriate volume of dichloromethane. Ethanol was then added to this mixture, which was centrifuged at 8000 rpm for 15 min to remove the carrier material. The supernatant was transferred to PBS and reacted with 75% sulfuric acid. The absorbance was measured at 482 nm to quantify the AZM content in the supernatant. All experiments were conducted in triplicates. The drug loading and encapsulation efficiencies of the AZM@PLGA-SF microspheres were calculated using Equations (1) and (2):

(1) Drug loading=(weight of drug in the microspheres/weight of microspheres)×100%

 

(2) Encapsulation efficiency=(drug loading/theoretic drug loading)×100%

### AZM release from AZM@PLGA-SF microspheres

Briefly, sample microspheres (2 mg) were placed in a dialysis bag (MW, 3500) containing 2 ml of an ethanol/PBS mixture (*v*/*v* = 1:4). The dialysis bag was then suspended in 40 ml of the same medium. The assembly was placed in a shaking incubator at 37°C and 100 rpm. At predetermined time intervals (0.5, 1, 2, 4, 6, 8, 10, 12, 24, 72, 120, 144, and 168 h), 4 ml of medium was withdrawn and replaced with an equal volume of fresh medium. Each sample of the withdrawn medium was mixed with 4 ml 75% (v/v) sulfuric acid and allowed to react for 30 min. The concentration of the released AZM was subsequently quantified using a UV–visible spectrophotometer (PerkinElmer Lambda 35) at a wavelength of 482 nm. The cumulative AZM release (%) was calculated using Equation (3) [30]:

(3) $$Q\left(\%\right)=\frac{V_{0}\times Cn+V\times \sum_{i=1}^{n-1}C_{i}}{m}\times 100\%\;$$

where *Q* is the cumulative AZM release (%); *V* is the volume that was withdrawn (4 ml); *C_i_* is the concentration at time *i* (*i* = *n* − 1), mg/ml; *V*_0_ is the total volume of ethanol/PBS mixture outside the dialysis bag (40 ml); *Cn* is the concentration at the selected time, mg/ml and *m* is the total milligram amount of AZM in AZM@PLGA-SF microspheres, mg.

Subsequently, the mechanism of drug release was analyzed using various release models (zero order, first order, Higuchi, Weibull, Ritger–Peppas) to understand the release kinetics of AZM from PLGA microspheres [26].

### Rat model of periodontitis

All animal experiments were approved by the Animal Welfare and Ethics Committee (Approval No.: IRM-DWLL-2019036) of the Experimental Animal Center at the Institute of Radiation Medicine, Chinese Academy of Medical Sciences. A total of 36 male Sprague-Dawley rats (8-wk-old; 220–250 g) were acquired from Beijing Vital River Laboratory Animal Technology Co., Ltd. After 1 wk adaptation period, 27 rats were selected for periodontitis induction, using a previously detailed methodology [31]. Briefly, rats were anesthetized with pentobarbital sodium (50 mg/kg). Orthodontic ligature wires were tied around the cervical region of the maxillary first molars (M1) on both sides. Subsequently, the rats were provided with a diet comprising 10% (w/v) sugar water and sticky feed to accelerate periodontitis establishment. Nine rats were assigned to the normal group and did not receive any treatment. After 8 wk, three rats from each group were randomly selected to confirm the development of the periodontitis model.

### Treatment schedules

After successful validation of the periodontitis model, the ligatures were removed from the remaining 24 rats in the model group. Subsequently, the rats were randomly divided into four treatment groups (saline, PLGA-SF, AZM and AZM@PLGA-SF; *n* = 6/group). We selected dosage of AZM according to a clinical study which used 0.2 ml prepared gel which contained 0.5% AZM injected into periodontal pockets [32]. And there is a simple practice guide for dose conversion between animals and human [33]. Bilateral injections were administered into the palatal gingival tissue of maxillary M1 of each rat. In the saline group, 0.05 ml of saline was injected on each side of the maxillary M1. The PLGA-SF group was administered 0.05 ml injections of a PLGA-SF microsphere suspension (devoid of AZM) on each side. The AZM group was administered 0.05 ml of the raw drug AZM on each side, with each injection containing 0.05 mg of AZM [34]. Similarly, the AZM@PLGA-SF group received bilateral injections of 0.05 ml of the AZM@PLGA-SF microsphere (0.05 mg AZM). The normal group received no drug intervention. Finally, the rats were euthanized 6 wk post-treatment.

### Assessment of periodontal parameters

Periodontal parameters, including sulcus bleeding index (SBI), gingival index (GI) and probing depth (PD), were assessed at the palatal sites of the maxillary M1 and second molars (M2) in rats according to methods outlined previously [31]. Following successful induction of periodontitis (end of wk 8), baseline measurements were recorded immediately commencing drug administration. Subsequent assessments were performed biweekly. An experienced practitioner performed all measurements in triplicate to ensure consistent and reliable measurements.

### Analysis of inflammatory cytokines in gingival crevicular fluid (GCF)

GCF samples were collected from the palatal aspect of the maxillary M1. Initially, the designated area was cleaned and air-dried. Absorbent paper points (Size 25#) were delicately inserted into both the mesial and distal gingival sulci of the molars. These points were then transferred to centrifuge tubes pre-filled with 100 µl of PBS solution [35]. This collection process was repeated thrice to ensure an adequate sample volume. Following collection, the concentrations of various inflammatory cytokines, namely, tumor necrosis factor (TNF)-α, interleukin (IL)-6, interferon (IFN)-γ, IL-2, IL-17A, IL-4 and IL-10 were measured within the GCF. This analysis was performed using the Rat Customs 7-Plex Kit (QuantoBio, Beijing, China), which employs high-throughput technology for multifactorial cytokine detection.

### Hematoxylin–eosin (H&E) staining of periodontal tissues

The left maxillary specimens were promptly fixed in 4% paraformaldehyde for 48 h, followed by perfusion with ethylenediaminetetraacetic acid (pH 7.2; Solarbio, Beijing, China) for 4 wk. After dehydration, the specimens were embedded in paraffin. Blocks were then oriented parallel to the sagittal axis, and serial sections of 5 µm thickness were prepared. The region of interest (ROI) was defined as the interproximal area between M1 and M2. The sections were stained with H&E (Solarbio, Beijing, China) and examined under a light microscope to identify any evidence of periodontal tissue destruction and regeneration. A semi-quantitative scoring system was used to assess the inflammatory cell infiltration in the affected gingival tissue. Within this scoring framework, a score of 0 was negative; 1 indicated less than 30% inflammatory cells; 2 denoted 30%–60% inflammatory cells and 3 indicated >60% inflammatory cells within the evaluated area [36].

### Assessment of maxillary alveolar bone morphometry

Alveolar bone destruction and regeneration were evaluated using micro-CT and SEM techniques. Soft tissue residues on the right maxillary samples were removed using a 3% sodium hypochlorite solution. Subsequently, the samples were analyzed using a micro-CT scanner (SkyScan 1276, Bruker, Belgium) with a voxel size of 9 µm and X-ray energy of 55 kV and 100 µA. Three-dimensional (3D) reconstructions were generated using SkyScan NRecon and SkyScan DataViewer software. The lengths between the lowest point of the crown enamel and the non-discrete bone height of the target root (ABLs, µm) and the length from the lowest point of the crown enamel to the lowest point of the target root apex (RBLs, µm) were measured to calculate the alveolar bone absorption ratio (ABLs/RBLs) [37]. For histomorphometric analysis, sections were obtained using the SkyScan DataViewer. A polygonal ROI (excluding roots) was identified between M1 and M2. The ROI was determined from the distopalatal side of M1 to the mesiopalatal side of M2 (length), 200 slices below the cementoenamel junction (CEJ) of M1 and M2 (height), and from the palatal side to the buccal side of M1 and M2 (width) [13, 36]. Trabecular microstructural characteristics, including bone volume/tissue volume (BV**/**TV, %), trabecular number (Tb.N, mm^−1^), trabecular thickness (Tb.Th, mm), trabecular separation (Tb.Sp, µm) and bone mineral density (BMD, mg**/**cm^3^), were analyzed using CTAn software (SkyScan). Following the micro-CT analysis, the samples were sequentially dehydrated in a graded ethanol series and coated with gold for SEM analysis [38]. The average distances from the CEJ to the alveolar bone crest (CEJ–ABC) in SEM images were quantitatively analyzed using Nano Measurer software. A longer distance from the CEJ to ABC suggests greater bone loss.

### Biological safety evaluation

The cytotoxicity of AZM@PLGA-SF microspheres to human periodontal ligament stem cells (hPDLSCs) was evaluated using the Cell Counting Kit-8 (CCK-8, Solarbio). The culture and identification of hPDLSCs were previously detailed in the literatures [31, 39]. Teeth were obtained from the Hospital of Stomatology Tianjin Medical University and were approved by the Ethics Committee (TMUhMEC20240505). Informed consent was obtained prior to the extraction of hPDLSCs from orthodontic teeth or caries-free third molars from patients aged 12–24 years. Flow cytometry was employed to confirm hPDLSC identity using antibodies against CD45, CD90 and CD146. *In vivo* study, the body weights of the rats were monitored and recorded biweekly throughout the treatment period. Blood biochemistry analyses and H&E staining of major organs (heart, liver, spleen, lung and kidney) were conducted to assess the biological safety of the AZM@PLGA-SF microspheres. Liver and renal function indices were measured using an automatic biochemical analyzer (Rayto, China).

### Statistical analysis

Statistical analyses were performed using SPSS version 26.0 (SPSS Corp., Armonk, NY, USA). Normally distributed continuous variables are represented as the mean ± SD. The statistical significance between the two independent groups was determined using a two-tailed unpaired Student’s *t*-test. For analyses involving more than two groups, one-way analysis of variance (ANOVA) was performed, followed by Tukey’s *post hoc* test. Statistical significance was defined as *P *<* *0.05. Graphical data were plotted using GraphPad Prism 8.0 (GraphPad Software, Inc., La Jolla, CA, USA).

## Results and discussion

### Characterization of AZM@PLGA-SF microspheres

AZM@PLGA-SF microspheres were synthesized using a single emulsion-solvent evaporation technique, followed by a layer-by-layer deposition approach. LbL assembly is an attractive method to construct various carriers or modify surface in biomedical field [27, 40]. The basic principles are electrostatic attractions and other weak interactions between the alternate polyelectrolytes. Both PLGA microspheres and SF possess negative charges. To facilitate the electrostatic-driven LbL assembly, poly(allylamine hydrochloride) (PAH), a positively charged pharmaceutical intermediate, was introduced into the system.

**Figure 2.**
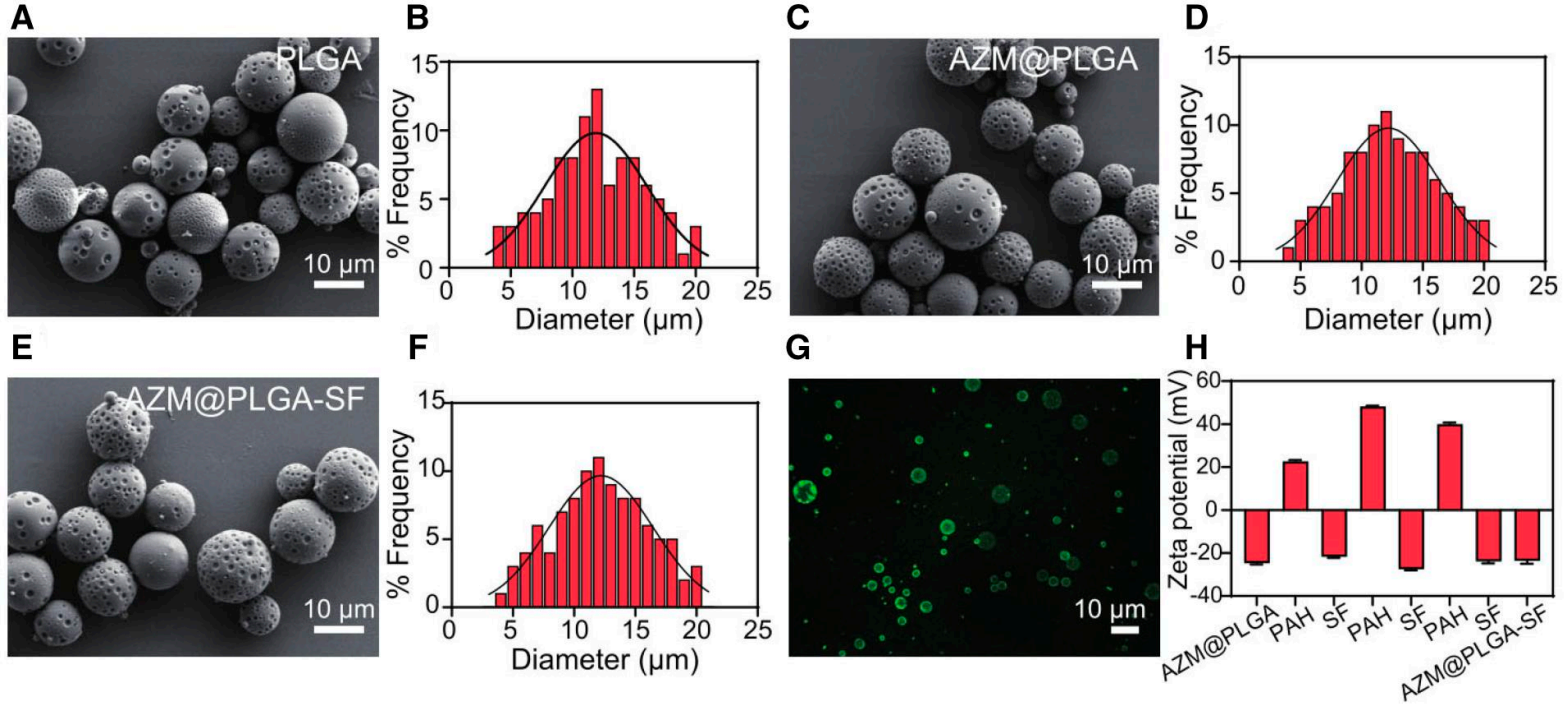
Characterization of AZM@PLGA-SF microspheres. (**A**) SEM image and size distribution of (**A**) blank PLGA microspheres, (**B**) AZM@PLGA microspheres and (**C**) AZM@PLGA-SF microspheres. (**D**) CLSM images of AZM@PLGA-SF microspheres with FITC-labeled PAH. (**E**) Evolution of zeta potential during deposition of the (PAH/SF)_3_ multilayer on AZM@PLGA microspheres (*n* = 3). AZM, azithromycin; PLGA, poly(lactic-co-glycolic acid); SF, silk fibroin; AZM@PLGA-SF microspheres, azithromycin-loaded PLGA microspheres coated with silk fibroin; CLSM, confocal laser-scanning microscope; FITC, fluorescein isothiocyanate; PAH, polyallylamine hydrochloride.

As shown in Supplementary Figure S1, raw AZM showed irregular shapes with varied grain size. The prepared PLGA microspheres displayed a spherical morphology characterized by dimpled surfaces (Figure 2A), with an average diameter of about 11.9 µm (Figure 2B). The AZM@PLGA microspheres had similar morphological properties (Figure 2C) and sizes to those of the PLGA microspheres, with an average diameter of 12.0 µm (Figure 2D). After the SF coating process, the morphology of AZM@PLGA-SF microspheres was consistent with that of the AZM@PLGA microspheres (Figure 2E), with an average diameter of about 12.5 µm (Figure 2F). Reportedly, microspheres with dimpled surfaces enhance cellular adhesion [26]. Additionally, studies have reported that microspheres sized 10–100 µm size can serve as intramuscular or subcutaneous depots, as they are small enough for syringe injection yet large enough to avoid uptake by phagocytic cells [41–43]. The results present in Supplementary Figure S2A and B demonstrated that both AZM@PLGA microspheres and AZM@PLGA-SF microspheres could pass through the 34G (0.18 × 8 mm) narrow needle. It guarantees minimal invasiveness and the direct administration of the drug in the targeted site.

Next, we performed XRD and FTIR to investigate AZM physical state in PLGA microspheres and after SF modification. As shown in Supplementary Figure S3A, raw AZM displayed several sharp and narrow diffraction peaks at 7.92°, 9.82°, 12.02°, 12.94°, 16.34°, 15.56°, 16.36°, 17.46°, 18.66°, 19.78°, 20.86°, and 23.96°. While, no aforementioned diffraction patterns were observed in AZM@PLGA microspheres and AZM@PLGA-SF microspheres. Additionally, as shown in Supplementary Figure S3B, raw AZM showed clear absorption peaks at 3496, 1282, 1269 cm^−1^ and so on. Both AZM@PLGA microspheres and AZM@PLGA-SF microspheres exhibited a clear absorption peak at 2950 cm^−1^, one characteristic absorption peaks of PLGA. Notably, the sharp high-frequency peak at 3496 cm^−1^ was no longer detected. Instead, broad bands in the range of 3300–3600 cm^−1^ were observed in both AZM@PLGA microspheres and AZM@PLGA-SF microspheres. In comparison to AZM@PLGA microspheres, AZM@PLGA-SF microspheres displayed a band at 1640 cm^−1^, corresponding to the amide I peak of SF. These findings collectively suggested that AZM was not simply physically mixed with PLGA but was instead encapsulated into the PLGA microspheres. SF was successfully deposited on the surface of these microspheres.

Subsequently, FITC fluorescence labeling of PAH further confirmed the layering of SF and PAH on the AZM@PLGA microsphere surface. Laser confocal microscopy revealed visible green fluorescence on the surface of AZM@PLGA-SF microspheres, confirming the successful deposition of SF (Figure 2G). Furthermore, variations in the zeta potential throughout the surface modification process were evaluated meticulously. The zeta potential of the AZM@PLGA microspheres was −25.07 mV. After alternating deposition with positively charged PAH and negatively charged SF, the potentials were 23.02, −22.17, 48.57, −26.95, 40.62, and −23.47 mV, respectively, and finally stabilized at −23.77 mV (Figure 2H). These results confirmed the successful deposition of intermediate layers of PAH and SF on the surface of AZM@PLGA microspheres. Considering the documented positive charge of inflamed periodontal tissues [44], the development of negatively charged AZM@PLGA-SF microspheres represents a targeted strategy for treating periodontal inflammation. This attribute substantially enhances the potential for electrostatic interactions with positively charged inflamed tissues, rendering these microspheres particularly suitable for localized therapy in periodontitis.

### Drug loading, encapsulation efficiency and release profile of AZM@PLGA-SF microspheres

**Figure 3.**
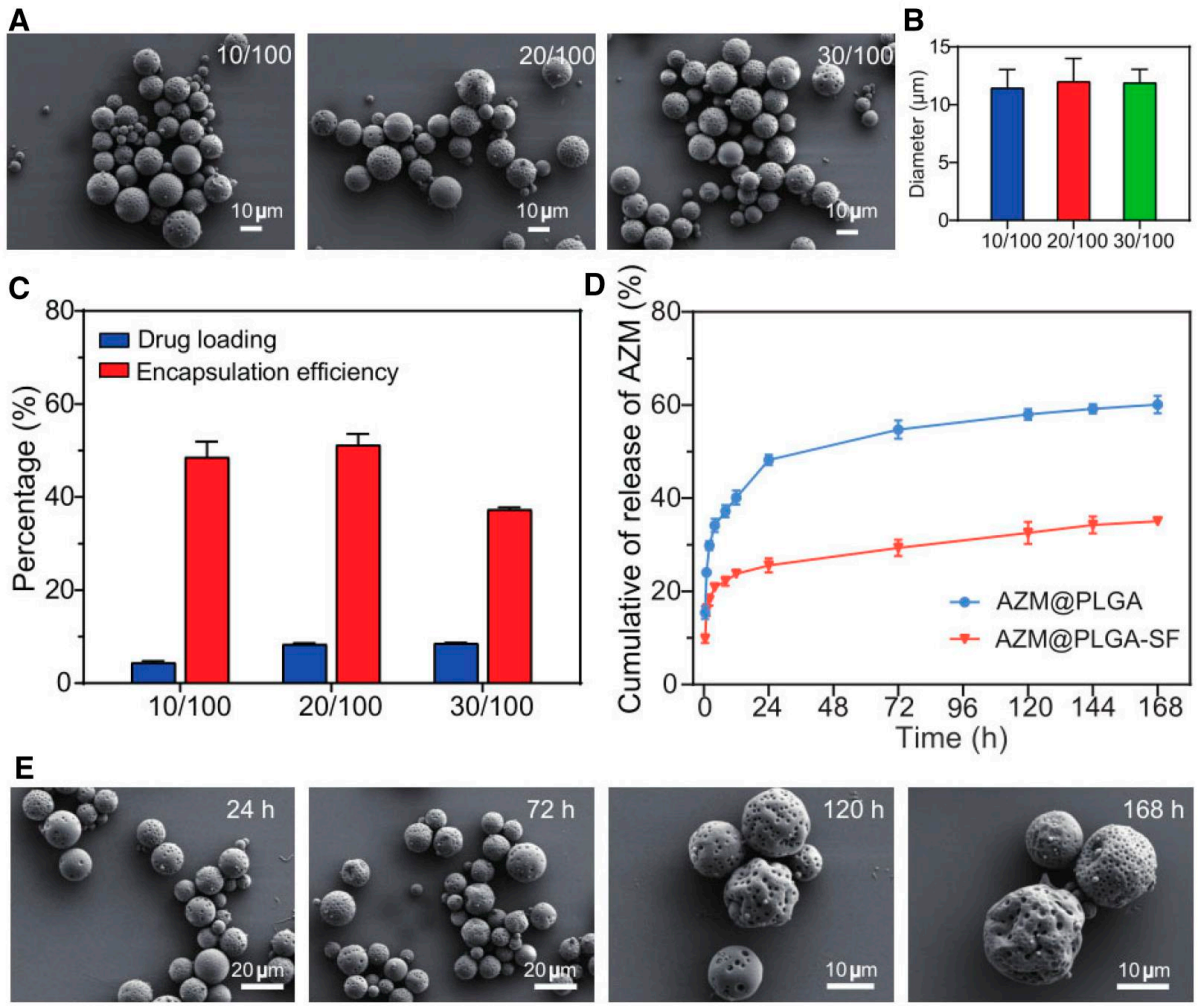
Drug loading, encapsulation efficiency and AZM release profiles of AZM@PLGA-SF microspheres. (**A**) SEM images, (**B**) size distribution and (**C**) drug loading and encapsulation efficiency of AZM@PLGA-SF microspheres with different AZM/PLGA ratios. (**D**) AZM release profiles of AZM@PLGA and AZM@PLGA-SF microspheres. (**E**) SEM images of AZM@PLGA-SF microspheres after AZM release at different time points. Data are presented as mean ± SD (*n* = 3). AZM, azithromycin; PLGA, poly(lactic-co-glycolic acid); SF, silk fibroin; AZM@PLGA-SF microspheres, azithromycin-loaded PLGA microspheres coated with silk fibroin; SEM, scanning electron microscopy.

The effects of the AZM-to-PLGA feed ratio on the morphology, size, drug loading and encapsulation efficiency of AZM@PLGA-SF microspheres were explored. No significant morphological differences were detected between microspheres prepared using varying formulation ratios (Figure 3A). Particle size evaluations revealed average diameters of 11.5, 12.0 and 11.9 µm for AZM/PLGA feed ratios of 10/100, 20/100 and 30/100, respectively, indicating no significant size variance across these ratios (Figure 3B). The drug loading efficiencies were calculated to be 4.4%, 8.4% and 8.6% for the respective feed ratios, whereas the encapsulation efficiencies were 48.6%, 51.2% and 37.4%, respectively (Figure 3C). The observed trends are consistent with those reported previously [45]. Therefore, it is important to select an effective drug delivery ratio to avoid drug wastage. Accordingly, we selected the 20/100 AZM/PLGA ratio for further experiments.

Subsequently, we assessed the release of AZM from AZM@PLGA and AZM@PLGA-SF microspheres. Within the first 24 h, the AZM release rate from the AZM@PLGA and AZM@PLGA-SF microspheres was 48.21% and 25.56%, respectively. Over 168 h, the cumulative AZM release reached 60.10% from the AZM@PLGA microspheres and 35.07% from the AZM@PLGA-SF microspheres, indicating that AZM was slowly released from the SF-modified microspheres (Figure 3D). Subsequent post-release SEM analysis revealed no significant morphological changes in the AZM@PLGA-SF microspheres within the first 24 h, with notable surface contraction detected at 72 h. After 120 h, the microspheres exhibited a depressed appearance, although spherical integrity was maintained. After 168 h, the AZM@PLGA-SF microspheres displayed a wrinkled surface without pores (Figure 3E). In contrast, the AZM@PLGA microspheres exhibited some porous and collapsed structure (Supplementary Figure S4). This may be explained by the slower degradation rate of SF compared to PLGA.

In order to explore the mechanism of drug release, we fitted AZM release profiles using several release models, including zero order, first order, Higuchi, Weibull, Ritger–Peppas [26, 46]. We found that AZM@PLGA microspheres and AZM@PLGA-SF microspheres could be better agreed with Weibull and Higuchi equations, respectively. Subsequently, the Ritger–Peppas equation was applied due to the swellable nature of the PLGA matrix in ethanol. As shown in Table 1, the release of AZM from both AZM@PLGA microspheres (*n* = 0.177) and AZM@PLGA-SF microspheres (*n* = 0.160) followed Fickian diffusion. This suggests that drug release in the ethanol system is primarily driven by the concentration gradient between the loaded drug and the surrounding release medium. Taken together, these findings elucidate the sustained release capabilities imparted by the SF coating, which likely acts as a barrier layer, preserving the microsphere structure and moderating AZM release. This barrier effect, attributed to the SF coating, is crucial for enhancing the safety and bioavailability of encapsulated drugs.

**Table 1.** Release models of AZM@PLGA and AZM@PLGA-SF microspheres

	AZM@PLGA	AZM@PLGA-SF
Zero order	*y* = 0.203*x* + 31.639 *R* ^2^ = 0.699	*y* = 0.110*x* + 18.750 *R* ^2^ = 0.755
First order	*y* = 52.003(1 − e^−0.351^^*t*^) *R* ^2^ = 0.717	*y* = 28.689(1 − e^−0.546^^*t*^) R^2^=0.646
Higuchi	*y* = 3.041*t*^0.5^ + 25.129 *R* ^2^ = 0.855	*y* = 1.611*t*^0.5^+15.420 *R* ^2^ = 0.877
Weibull	*y* = 83.21(1 − e^(0.018*(^^*x*^^−0.043)^)^0.24^ *R* ^2^ = 0.995	–
Ritger−Peppas	*y* = 25.139*t^n^* *R* ^2^=0.962 *n* = 0.177 ± 0.013	*y* = 15.348*t^n^* *R* ^2^ = 0.961 *n* = 0.160 ± 0.011

### Establishment of an experimental periodontitis model *in vivo*

**Figure 4.**
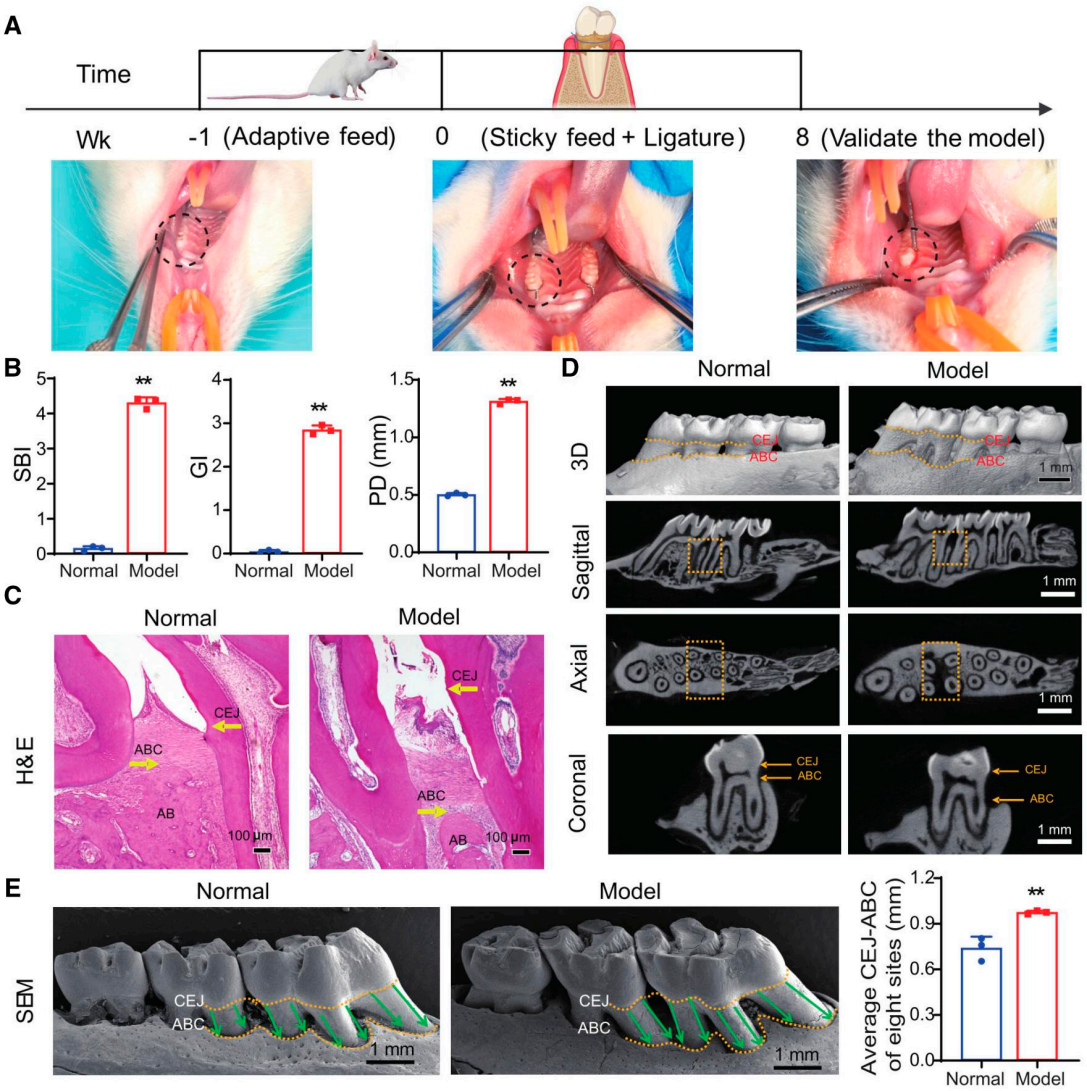
Experimental periodontitis model establishment. (**A**) Scheme illustrating the procedure of experimental periodontitis induction in rats; the intraoral photographs of rats’ maxillary first molar area 1 wk before model establishment, at the time of model establishment and before model establishment. (**B**) Assessment of periodontal parameters between two groups at wk 8: SBI, GI and PD. (**C**) At wk 8, H&E-stained images of the left maxillary alveolar bone of two rat groups. (**D**) 3D reconstruction images of the buccal view of the right maxillary alveolar bone of rats and three cross-sectional micro-CT images; the dotted lines and arrows represent CEJ and ABC, and the dotted boxes represent the ROI. (**E**) Quantitative analysis of the CEJ-ABC distance based on SEM images of the palatal view of the right maxillary alveolar bone of rats, with the dotted lines representing CEJ and ABC and the arrows representing the distance from CEJ to ABC. Data are presented as mean ± SD (*n* = 3), *P <* *0.05, **P <* *0.01. SBI, sulcus bleeding index; GI, gingival index; PD, probing depth; H&E, hematoxylin-eosin; micro-CT, microcomputed tomography; 3D, three-dimensional; SEM, scanning electron microscopy; AB, alveolar bone; ABC, alveolar bone crest; CEJ, cementoenamel junction. ROI, region of interest.

To investigate the therapeutic efficacy of the AZM@PLGA-SF microspheres in periodontitis *in vivo*, we established an experimental periodontitis rat model using a ligature-induced method combined with a sticky diet. Initially, following 1 wk adaptive feeding period, the gingival conditions of the rat maxillary first molars (M1), including color, shape and texture, were observed to determine whether they were within the normal range prior to ligation. Subsequently, the ligatures were tied bilaterally around the cervical margins of the maxillary M1, and the rats were maintained on a sticky diet for 8 wk. After ligature removal and resuming the standard diet, notable symptoms of periodontitis, including gingival redness, swelling and substantial bleeding upon probing, were observed at the ligated sites of first molar and mesial of second molars (M2) (Figure 4A).

To quantitatively assess the inflammatory status within periodontal tissues, three periodontal parameters (sulcus bleeding Index [SBI], gingival index [GI] and probing depth [PD]) were evaluated. Both SBI and GI values of rats in the model group were significantly elevated, representing severity inflammation, when compared with those in the normal group (*P < *0.01). The mean PD value reached 1.31 mm in the model group, markedly surpassing the 0.50 mm observed in the normal group (*P *<* *0.01; Figure 4B). These data underscore the successful induction of periodontal tissue inflammation and subsequent connective tissue degradation in the ligated regions of rats, culminating in the formation of deep periodontal pockets.

Further histological examination using H&E staining revealed significant differences between rats in the model and normal groups. In the model group, rats exhibited epithelial ulceration within the gingival sulcus, pronounced expansion of the periodontal space, disorganized arrangement of periodontal ligament fibers, diminished alveolar crest height and increased distance between the CEJ and the alveolar bone crest (ABC) (Figure 4C). These observations confirmed the presence of both soft and hard periodontal tissue damage in the model group. Additionally, microcomputed tomography (micro-CT) provides a detailed assessment of alveolar bone destruction. Three-dimensional (3D) reconstructions and various cross-sectional images delineated marked bone resorption in the alveolar bone surrounding the upper M1 in the model group, particularly in the distal M1 and mesial aspect of M2 (region of interest, ROI) (Figure 4D). SEM images revealed that the CEJ–ABC distance was 0.98 mm in the model group, which was significantly higher than the 0.74 mm in the normal group (*P *<* *0.01) when assessed at eight designated points on the palatal aspect of the maxillary ligated region (Figure 4E). Collectively, these results conclusively demonstrated the successful establishment of an experimental periodontitis model over an 8-wk period.

### Efficacy of AZM@PLGA-SF microspheres in ameliorating inflammation *in vivo*

**Figure 5.**
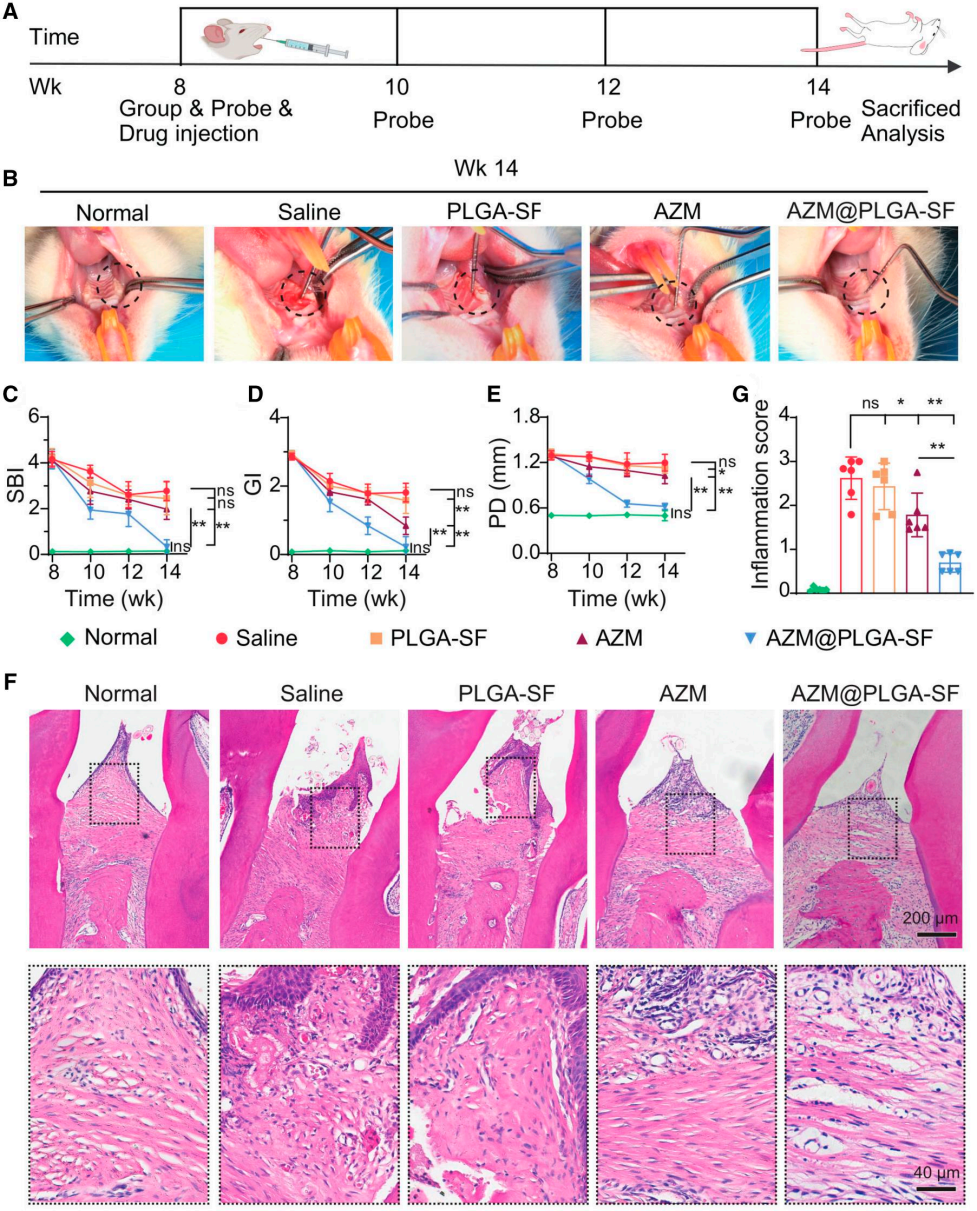
Therapeutic outcomes of AZM@PLGA-SF microspheres in a rat model of periodontitis. (**A**) Schematic diagram of the experimental treatment of a rat model of periodontitis model. (**B**) Intraoral photographs of rat maxillary first molars’ periodontal tissue after drug administration at wk 14. Analysis of (**C**) SBI, (**D**) GI and (**E**) PD of five groups at baseline, 10, 12 and 14 wk. (**F**) Histological analysis of papillary connective tissue between M1 and M2 after 6 wk of treatment. Representative images of H&E-stained tissue sections of papillary connective tissue between M1 and M2. (**G**) Semi-quantitative evaluation of inflammatory cell presence. Data are presented as mean ± SD (*n* = 6). *P <* *0.05, **P <* *0.01, ns: not significant. AZM, azithromycin; PLGA, poly(lactic-co-glycolic acid); SF, silk fibroin; AZM@PLGA-SF microspheres, azithromycin-loaded PLGA microspheres coated with silk fibroin; H&E, hematoxylin-eosin; SBI, sulcus bleeding index; GI, gingival index; PD, probing depth; M1, first molar; M2, second molar; AB, alveolar bone; ABC, alveolar bone crest; CEJ, cementoenamel junction.

In this investigation, the efficacy of the AZM@PLGA-SF microspheres in treating periodontitis was assessed. Rats with induced periodontitis were randomly assigned to four treatment groups: saline, PLGA-SF, AZM and AZM@PLGA-SF. Periodontal parameters (SBI, GI and PD) were recorded biweekly to evaluate inflammation status (Figure 5A). Intraoral analysis at wk 14 revealed distinct variations in gingival inflammation among the treatment groups. The saline group exhibited persistent gingival inflammation characterized by pronounced redness, swelling and bleeding upon probing. Rats in the PLGA-SF-treated group displayed persistent signs of gingival inflammation, suggesting that the carrier material alone failed to mitigate this condition. Conversely, rats in the AZM group exhibited a reduction in gingival inflammation, indicating a moderate anti-inflammatory effect. Remarkably, in the AZM@PLGA-SF group, the gingiva closely adhered to the tooth surface, and the morphology of the gingival tissue normalized, with probing not eliciting bleeding, resembling the tissue architecture of the normal group (Figure 5B). This implies that treatment with AZM@PLGA-SF microspheres effectively alleviated periodontitis, reverting the gingival tissue morphology to its normal state. Furthermore, SBI, GI and PD, which indicate the severity of inflammation with high scores, gradually declined in both the AZM and AZM@PLGA-SF groups (Figure 5C–E). By wk 14, the AZM@PLGA-SF group demonstrated significantly lower SBI, GI and PD values than the saline group (*P *<* *0.01). Notably, the differences between the AZM@PLGA-SF and AZM groups were statistically significant (*P *<* *0.01), with values of the AZM@PLGA-SF group approaching those of the normal group (*P *>* *0.05).

Histological evaluation was performed by examining H&E-stained sections. The saline- and PLGA-SF-treated groups displayed compromised gingival epithelial barriers and numerous immune cells. The number of inflammatory cells was lower in the AZM group than in the saline group (*P *<* *0.05), along with a disordered arrangement of some fibroblasts. Notably, the AZM@PLGA-SF group exhibited significant improvements, with the lowest inflammatory cell score (*P *<* *0.01, compared to the saline group; *P *<* *0.01, compared to the AZM group) (Figure 5F, G). These findings suggest that AZM@PLGA-SF microspheres exhibit significantly enhanced therapeutic efficacy, nearly restoring gingival tissues to their normal health status. The superior performance of AZM@PLGA-SF microspheres can be attributed to the unique properties of the PLGA-SF matrix, which facilitates sustained release and enhances adhesion, preventing premature clearance of AZM by gingival crevicular fluid or saliva. This prolongs the AZM activity, thereby augmenting its anti-inflammatory effect.

### AZM@PLGA-SF microspheres modulate the inflammatory cytokines in GCF

The mechanisms through which the AZM@PLGA-SF microspheres modulate inflammation in periodontitis were explored. The GCF, a complex amalgam of physiological and inflammatory exudates from the gingival vasculature, serves as an essential reservoir of biomarkers indicative of periodontal tissue inflammation [47]. This fluid comprises a wide range of cytokines, including pro-inflammatory types (e.g. TNF-α, IL-6, IFN-γ, IL-2, IL-17A) and anti-inflammatory (e.g. IL-4, IL-10) cytokines, which are critical in maintaining the balance between homeostasis and inflammatory responses within tissue cells, lymphocytes and other related cellular types [47, 48]. Immune cells within periodontal tissues release pro-inflammatory cytokines such as TNF-α and IL-6, particularly under pathogenic stimuli, prompting the recruitment and activation of particular immune cell subsets and thus inflicting direct tissue damage [48]. Furthermore, cytokines linked with Th1 responses, including IFN-γ and IL-2, are known to drive cellular immune responses, exacerbating damage to periodontal cells and tissues during inflammatory episodes [49]. In addition, the Th17-related cytokine IL-17A, which is associated with the Th1 cytokine profile, further exacerbates periodontal inflammation [50]. Conversely, cytokines associated with the Th2 and Treg responses, such as IL-4 and IL-10, play crucial roles in protecting against unregulated inflammation [51, 52].

**Figure 6.**
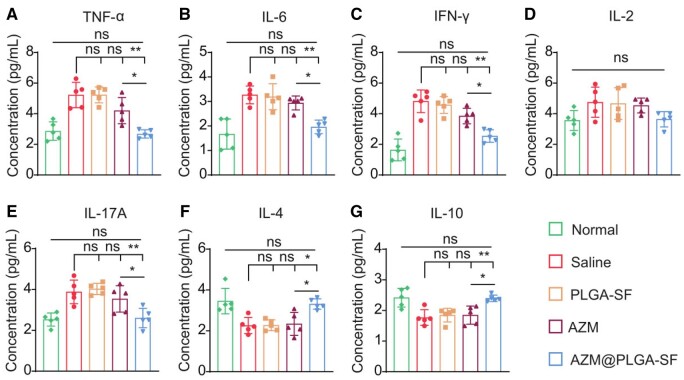
Quantification of inflammatory cytokines in the GCF of rats at 14 wk. (**A–E**) Analysis of pro-inflammatory factors tumor necrosis factor (TNF)-α, interleukin (IL)-6, interferon (IFN)-γ, IL-2 and IL-17A in the GCF. (**F**, **G**) Analysis of anti-inflammatory factors IL-4 and IL-10 in GCF. Data are presented as mean ± SD (*n* = 5), *P <* *0.05, **P <* *0.01, ns: not significant. AZM, azithromycin; PLGA, poly(lactic-co-glycolic acid); SF, silk fibroin; AZM@PLGA-SF microspheres, azithromycin-loaded PLGA microspheres coated with silk fibroin; GCF, gingival crevicular fluid.

AZM has been shown to attenuate inflammation by reducing pro-inflammatory cytokine production and promoting the release of anti-inflammatory cytokines, thereby rectifying the periodontitis inflammatory microenvironment [53]. To evaluate the effect of AZM@PLGA-SF microspheres on inflammatory cytokine levels, high-throughput multifactor detection technology was employed to measure the levels of seven inflammatory cytokines in the gingival sulcus of treated rats. The levels of pro-inflammatory cytokines (TNF-α, IL-6, IFN-γ and IL-17A) were significantly reduced in the AZM@PLGA-SF treatment group, which were notably lower than those observed in the saline (*P *<* *0.01) and AZM (*P *<* *0.05) groups, aligning closely with levels observed in the normal group. Although IL-2 levels remained constant across the four treatment groups, the lowest concentrations were detected in the AZM@PLGA-SF group, albeit without statistical significance (Figure 6A–E). Moreover, the concentrations of the anti-inflammatory cytokines IL-4 and IL-10 were significantly higher in the AZM@PLGA-SF group than in the saline and AZM groups (*P *<* *0.05), reaching levels comparable with those in the normal group (*P *<* *0.05) (Figure 6F, G). These findings highlight the dual function of AZM@PLGA-SF microspheres in suppressing pro-inflammatory cytokine production while simultaneously enhancing anti-inflammatory cytokine levels, thus surpassing the efficacy of AZM monotherapy. Consequently, the AZM@PLGA-SF microspheres significantly enhanced the anti-inflammatory potential of AZM, resulting in reduced levels of pro-inflammatory cytokine and elevated levels of anti-inflammatory cytokines in the gingival sulcus of rats with periodontitis. This dual action could effectively ameliorate the inflammatory microenvironment in periodontitis.

### AZM@PLGA-SF microspheres promote periodontal tissue regeneration

Next, we evaluated the regenerative capacity of the periodontal tissue in rats administering AZM@PLGA-SF microspheres. The role of inflammatory cytokines in the degradation and regeneration of periodontal tissues is well-documented [54–56]. For example, the pro-inflammatory cytokine TNF-α enhances osteoclastic activity and inhibits the osteogenic activity of mesenchymal stem cells and osteoblasts, resulting in alveolar bone loss and affecting periodontal tissue repair and regeneration [14, 57]. We have previously demonstrated that AZM promotes the osteogenic differentiation of human periodontal ligament stem cells in an inflammatory microenvironment, thus promoting the regeneration of periodontal tissue [14]. In the current study, we further explored the potential of local treatment with AZM for periodontal tissue repair and regeneration.

**Figure 7.**
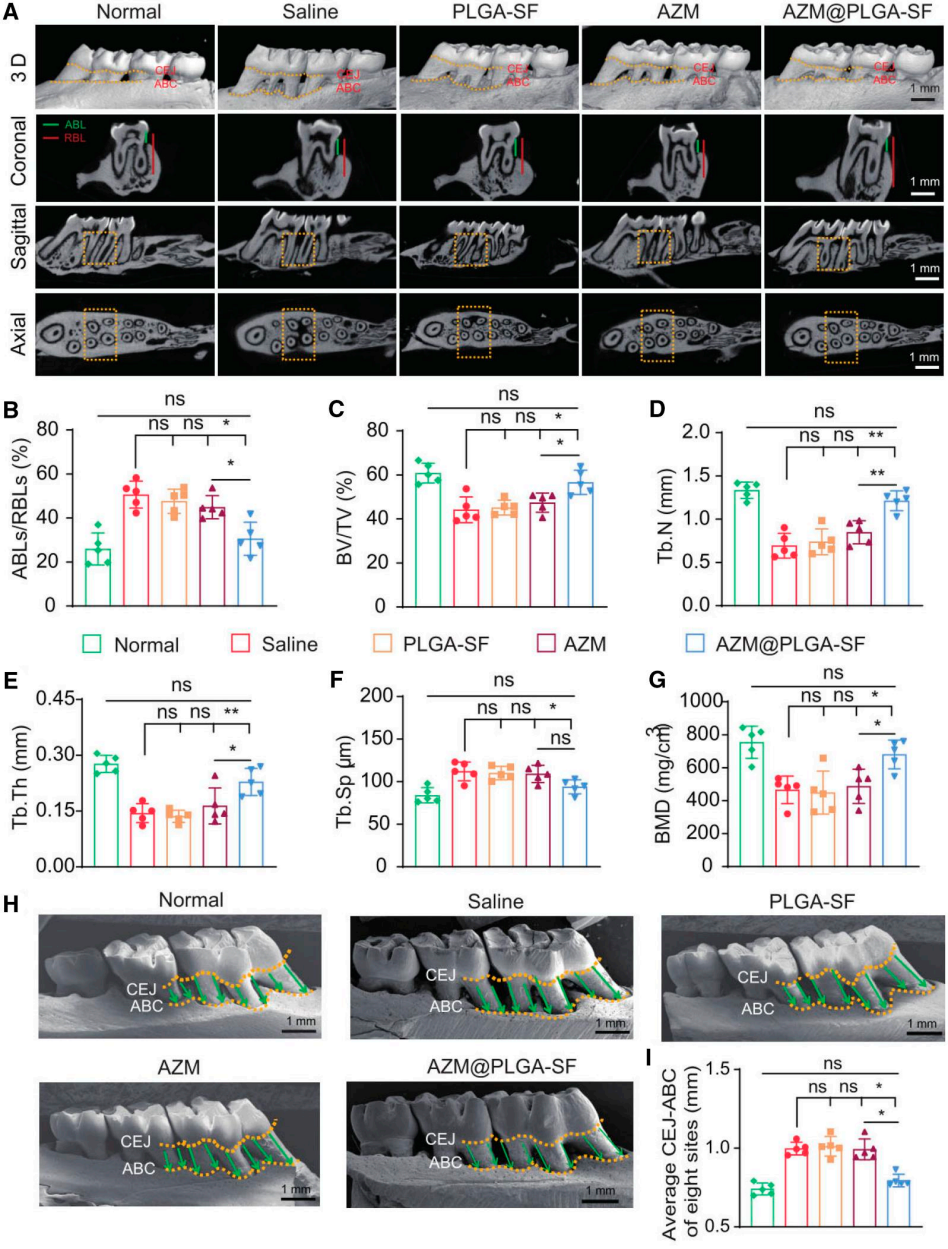
Effect of AZM@PLGA-SF microspheres on periodontal tissue regeneration. (**A**) Three-dimensional (3D) reconstruction images of the buccal view of the right maxillary alveolar bone of rats, and three cross-sectional micro-CT images, with the dotted lines representing CEJ and ABC, the solid lines representing ABL (short line) and RBL （long line), the dotted boxes representing the region of interest (ROI). (**B–G**) Analysis of micro-CT volumetric parameters: ABLs/RBLs, BV/TV, Tb.N, Tb.Th, Tb.Sp and BMD. (**H**) Quantitative analysis of CEJ–ABC distance based on SEM images of the palatal view of the right maxillary alveolar bone of rats, with the dotted lines representing CEJ and ABC and the arrows representing the distance from CEJ to ABC. Data are presented as mean ± SD, *P <* *0.05, **P <* *0.01, ns: not significant. AZM, azithromycin; PLGA, poly(lactic-co-glycolic acid); SF, silk fibroin; AZM@PLGA-SF microspheres, azithromycin-loaded PLGA microspheres coated with silk fibroin; Micro-CT, microcomputed tomography; SEM, scanning electron microscopy; CEJ, cementaoenamel junction; ABC, alveolar bone crest; ABL, the length between CEJ to alveolar bone; RBL, the length between CEJ to root bone area; BV/TV, bone volume/tissue volume; Th.N, trabecular number; Tb.Th, trabecular thickness; Tb.Sp, trabecular separation; BMD, bone mineral density.

Micro-CT analysis revealed notable differences among the treated groups. In the saline and PLGA-SF groups, alveolar bone resorption extended to the apical region of the maxillary ligature area. Conversely, the group treated with AZM alone displayed slight bone restoration. Remarkably, the group that received AZM@PLGA-SF microspheres exhibited substantial alveolar bone restoration (Figure 7A). Quantitative analysis revealed that the alveolar bone loss ratio (ABL/RBL) decreased significantly in the AZM@PLGA-SF group when compared with that in the saline and AZM groups (*P *<* *0.05), which was similar to that in the normal group (*P *>* *0.05) (Figure 7B). To further define the alveolar bone microstructure, a histomorphometric analysis of the ROI of the maxillary trabecular bones was performed. These parameters, BV/TV, Tb.N, Tb.Th and BMD, increased significantly, representing an increase in bone volume and bone mass, in the AZM@PLGA-SF group when compared to the saline and AZM groups. Meanwhile, Tb.Sp decreased remarkably, representing high bone mass, in the AZM@PLGA-SF group compared with the saline group. In addition, treatment with AZM@PLGA-SF microspheres recovered the microarchitectural parameters to levels observed in normal rats (Figure 7C–G). These findings suggest that the AZM@PLGA-SF microspheres promote alveolar bone repair and regeneration. Additionally, the AZM@PLGA-SF microsphere-treated rats demonstrated the shortest average CEJ to ABC distance when compared with the saline and AZM groups (*P *<* *0.01), with measurements approaching those observed in the normal group (*P* > 0.05), as determined by SEM (Figure 7H, I). These findings underscore the potent *in vivo* therapeutic efficacy of the AZM@PLGA-SF microspheres against periodontitis in rats, substantially enhancing the repair and regeneration of periodontal tissues.

### Biological safety of AZM@PLGA-SF microspheres

**Figure 8.**
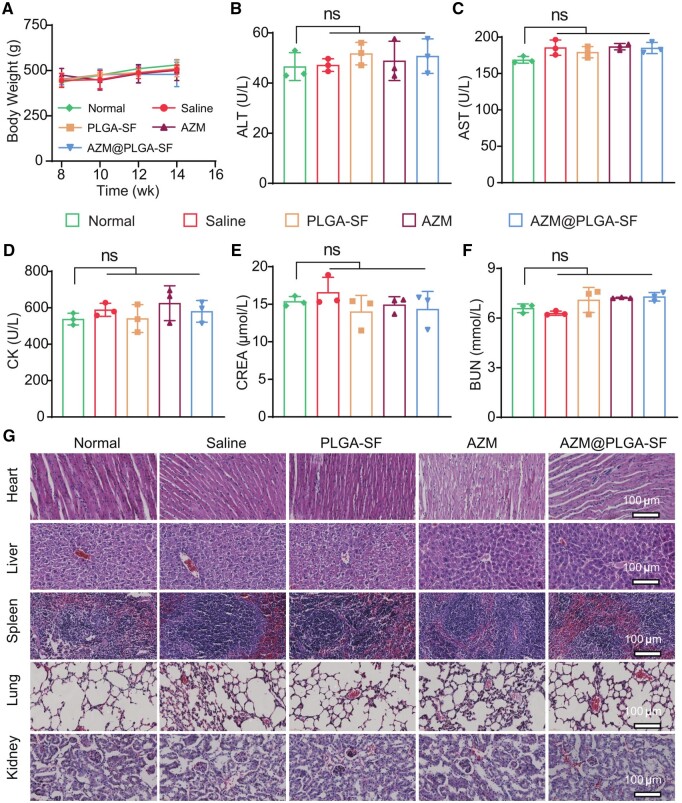
Biosafety of AZM@PLGA-SF microspheres *in vivo*. (**A**) Body weight changes in the five groups of rats. (**B–F**) Blood biochemistry analysis of liver function (ALT and AST), cardiac function (CK) and kidney function (CREA and BUN). (**G**) H&E-stained sections of the heart, liver, spleen, lung and kidney of rats sacrificed post-treatment. Data are presented as mean ± SD (*n* = 3); ns represents no significant difference. AZM, azithromycin; PLGA, poly(lactic-co-glycolic acid); SF, silk fibroin; AZM@PLGA-SF microspheres, azithromycin-loaded PLGA microspheres coated with silk fibroin; ALT, alanine aminotransferase; AST, aspartate aminotransferase; CK, creatine kinase; CREA, creatinine; BUN, blood urea nitrogen.

Human periodontal ligament stem cells (hPDLSCs) were chosen to evaluate the *in vitro* cytotoxicity of AZM@PLGA-SF microspheres using CCK-8 assay. First, hPDLSCs were isolated from healthy volunteers, cultured and identified. The specific biomarkers showed negative expression of CD45, positive expression of CD90 and CD146, indicating that the extracted cells were periodontal mesenchymal stem cells (Supplementary Figure S5). As shown Supplementary Figure S6, only PBS without microspheres were used as a negative control. AZM@PLGA-SF microspheres at concentrations ranging from 0 to 1000 µg/ml showed no significant cytotoxicity, revealing its good biocompatibility even at high concentrations (1000 µg/ml). Then, we performed an *in vivo* assessment of the biological safety of AZM@PLGA-SF microspheres. The body weight of experimental rats was monitored throughout the treatment period. The results revealed a continuous growth pattern with no significant differences among five groups (Figure 8A), indicating that the treatment did not adversely impact the general health of the animals. Subsequently, the liver, heart and renal functions were evaluated using blood biochemical analysis. Liver function was assessed by measuring serum alanine aminotransferase (ALT) and aspartate aminotransferase (AST) levels. These assessments revealed no significant differences among the groups, suggesting that the administration of AZM@PLGA-SF microspheres did not induce hepatic toxicity (Figure 8B, C). Likewise, cardiac health was evaluated by measuring creatine kinase (CK) levels, with the results demonstrating no significant variation among the groups (Figure 8D). Furthermore, renal function was assessed by evaluating blood urea nitrogen (BUN) and serum creatinine (CREA) levels, both markers of renal health. Consistent levels detected across all groups further substantiated the nontoxic nature of the materials on renal function (Figure 8E, F). Finally, critical organs, including the heart, liver, spleen, lungs and kidneys, harvested from each rat group were histologically examined. H&E staining revealed no observable changes, confirming the absence of adverse histological effects (Figure 8G). Collectively, these findings indicate that AZM@PLGA-SF microspheres are biologically safe for use in treating periodontitis.

## Conclusions

In summary, we successfully fabricated AZM@PLGA-SF microspheres for localized drug therapy in periodontitis. AZM@PLGA-SF microspheres effectively mitigated the initial burst release of AZM and increased its bioavailability at the target site. *In vivo*, treatment with AZM@PLGA-SF microspheres substantially reduced periodontal inflammation by reducing the expression of pro-inflammatory cytokines (TNF-α, IL-6, IFN-γ, IL-2 and IL-17A) while simultaneously enhancing the levels of pro-inflammatory cytokines (IL-4 and IL-10) in the gingival sulcus. This alteration in the inflammatory profile of the periodontal environment notably facilitates the repair and regeneration of damaged tissues, thereby restoring them to near-normal conditions. Moreover, the AZM@PLGA-SF microspheres demonstrated a high level of biological safety, indicating their potential for safe application in periodontal therapy. This study presents an anti-inflammatory strategy for treating periodontitis, which not only effectively ameliorates periodontal inflammation but also promotes periodontal tissue regeneration. This work offers considerable promise for future clinical applications in the management of periodontitis.

## Supplementary Material

rbae146_Supplementary_Data

## Data Availability

All data required to evaluate the conclusions in the paper are available in the article itself and/or the Supplementary data. Requests for materials related to this study should be directed to the corresponding author and obtained through an MTA.
