# Supplementary material for: Azithromycin-loaded PLGA microspheres coated with silk fibroin ameliorate inflammation and promote periodontal tissue regeneration
Source: Regen Biomater. 2024 Dec 14;12:rbae146. doi: 10.1093/rb/rbae146 (PMC11717352; doi:10.1093/rb/rbae146)
Supplement: rbae146_Supplementary_Data [file rbae146_supplementary_data.docx]

**Supplementary Information**

Azithromycin-loaded PLGA Microspheres Coated with Silk Fibroin Ameliorate Inflammation and Promote Periodontal Tissue Regeneration

Zhaoguang Ouyang^1,2,5,‡^, Xiaoyu Chen^1,2,‡^, Zhengyang Wang^1,2^, Yue Xu^1,2^, Zhe Deng^4,6^, Liangyu Xing^1,2^, Li Zhang^1,2^, Meilin Hu^1,2^, Haocong Li^1,2^, Tengye Lian^1,2^, Feng Gao^1,2^, Chunyi Liu^1,2^, Yangyang Zhou^1,2^, Lu Sun^7,8^, Ying ChengYao Wang^9,10^, Dayong Liu^1,2,3,^*

^1^Department of Endodontics, Tianjin Medical University School and Hospital of Stomatology & Tianjin Key Laboratory of Oral Soft and Hard Tissues Restoration and Regeneration, Heping District, Tianjin 300070, PR China

^2^Tianjin Medical University Institute of Stomatology, Heping District, Tianjin 300070, PR China

^3^School and Hospital of Stomatology, Hebei Medical University & Hebei Key Laboratory of Stomatology & Hebei Clinical Research Center for Oral Diseases, Shijiazhuang, Hebei, 050011, PR China

^4^ College of Integrated Chinese and Western Medicine, Hunan University of Chinese Medicine, Changsha, Hunan, 410208, PR China

^5^Department of Preventive Dentistry, School and Hospital of Stomatology, Guangdong Engineering Research Center of Oral Restoration and Reconstruction & Guangzhou Key Laboratory of Basic and Applied Research of Oral Regenerative Medicine, Guangzhou Medical University, Guangzhou, 510013, PR China

^6^Sidney Kimmel Comprehensive Cancer Center at Johns Hopkins, Baltimore, Maryland 21205, USA

^7^Department of Periodontics and Oral Medicine, University of Michigan School of Dentistry, Ann Arbor, Michigan, 48105, USA

^8^Periodontal and Implant Microsurgery Academy (PiMA), University of Michigan School of Dentistry, Ann Arbor, Michigan, 48105, USA

^9^Department of Operative Dentistry and Endodontics, Tianjin Stomatological Hospital, School of Medicine, Nankai University, Tianjin, 300041, PR China

^10^Tianjin Key Laboratory of Oral and Maxillofacial Function Reconstruction, Tianjin, 300041, PR China

* Correspondence addresses: [dyliuperio@tmu.edu.cn](mailto:dyliuperio@tmu.edu.cn)

‡ These authors contributed equally to this work.

**Supplementary Figures**

**Figure S1.** Scanning electron microscopy (SEM) image of raw Azithromycin (AZM).


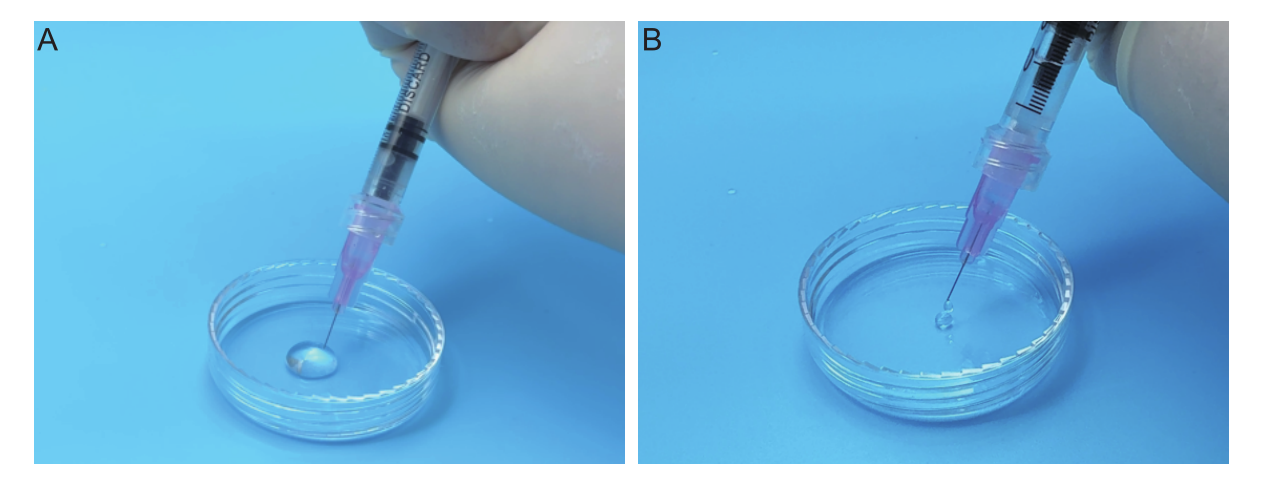


**Figure S2.** Digital pictures of AZM@PLGA (A) and AZM@PLGA-SF microspheres suspension (B) could pass through the 34G (0.18*8 mm) needle, respectively.


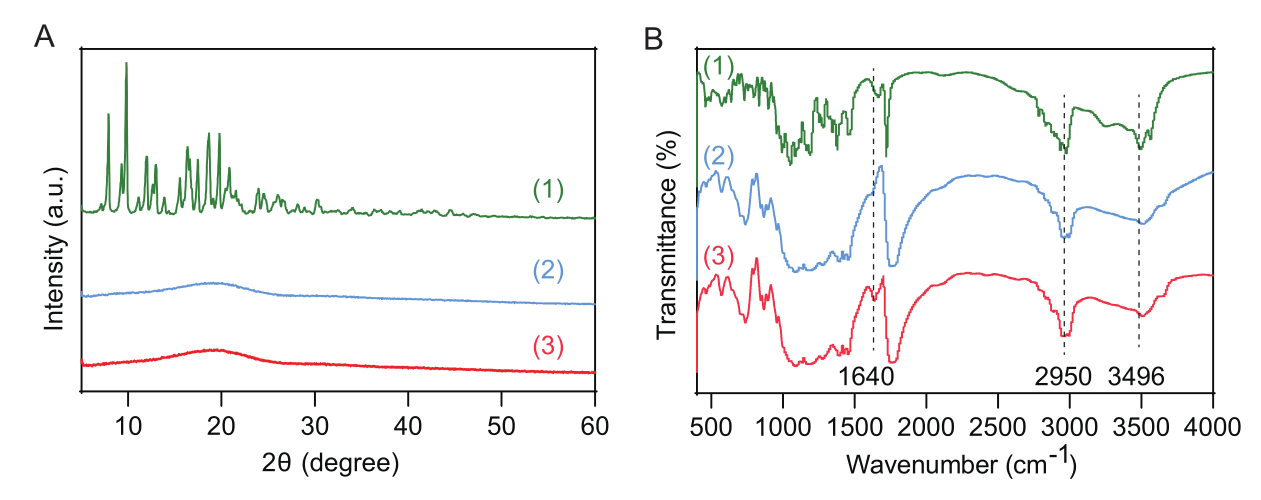


**Figure S3.** X-ray powder diffractometer (XRD) patterns (A) and Fourier transform infrared spectroscopy (FTIR) spectra (B) of raw AZM (1), AZM@PLGA microspheres (2), and AZM@PLGA-SF microspheres (3).

**Figure S4.** SEM image of AZM@PLGA microspheres after AZM release at 168 h (7 d).


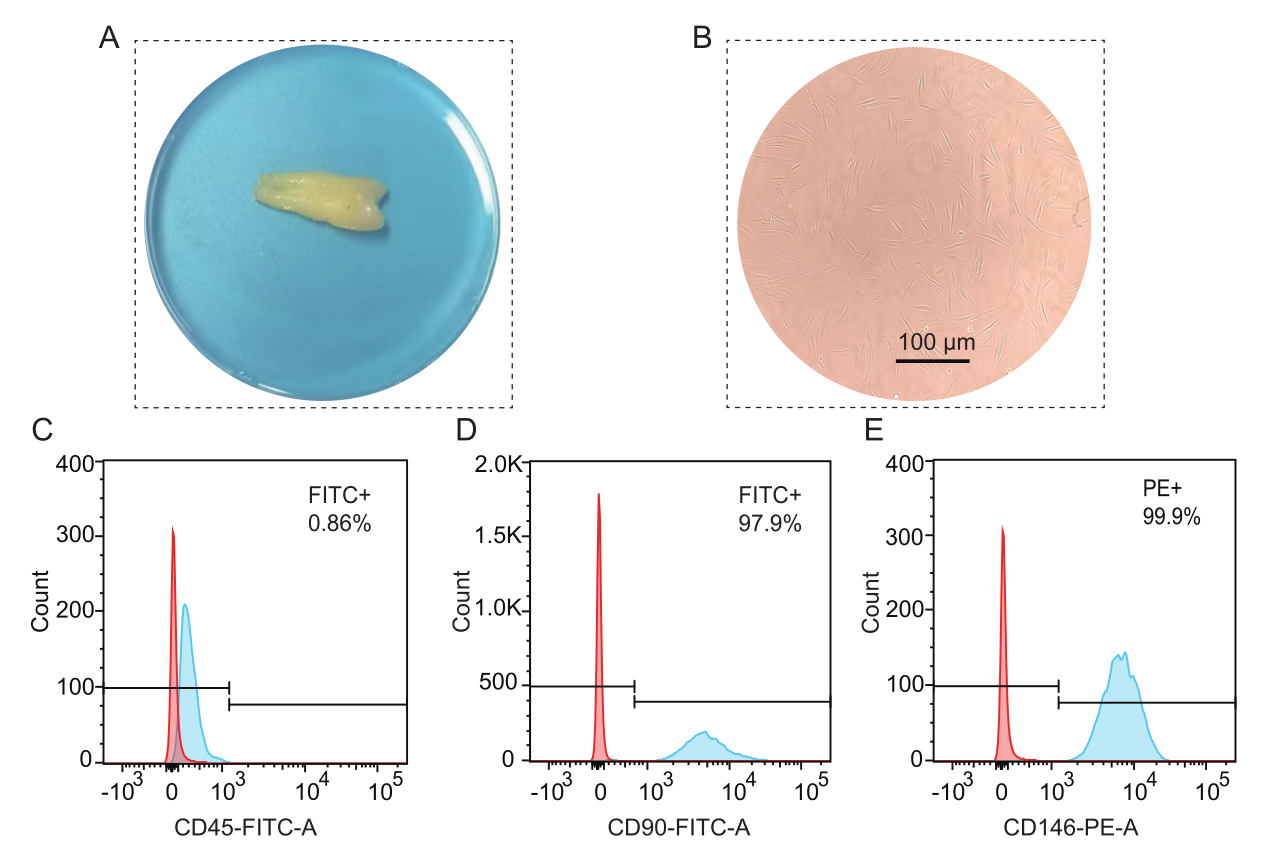


**Figure S5.** Primary culture and identification of human periodontal ligament stem cells (hPDLSCs). (A) Digital photograph of tooth. (B) Optical microscopy image of hPDLSCs (third-passage). (C-E) Flow cytometric analysis of hPDLSCs showed negative result for CD45, and positive expression of cell markers CD90, CD146.


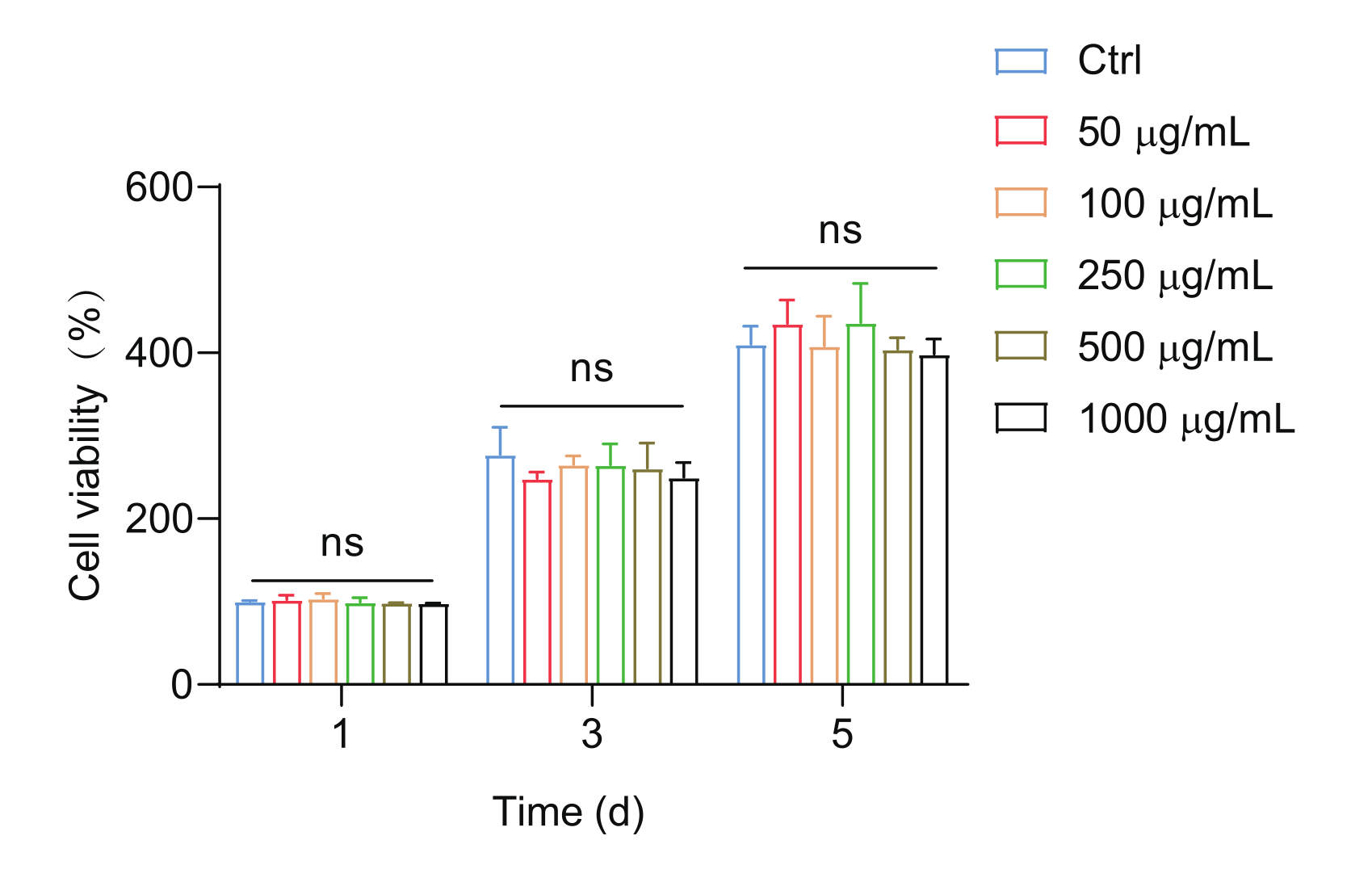


**Figure S6.** Cell viability of hPDLSCs exposed to AZM@PLGA-SF microspheres at concentrations ranging from 0 to 1000 μg/mL. Data are presented as mean ± standard deviation (n=3); ns represents no significant difference.
